# UST1/435: HON's Fourth Survey on the Usage of the Internet for Medical and Health Internet Purposes

**DOI:** 10.2196/jmir.1.suppl1.e127

**Published:** 1999-09-19

**Authors:** C Boyer, V Baujard, T Nater, JR Scherrer, R Appel

**Affiliations:** ^1^Health On the Net Foundation, GenevaSwitzerland

**Keywords:** Survey, Internet Assess, User needs, Surveying, Assessment

## Abstract

**Introduction:**

To remain competitive, the providers of medical and health-related information must continually adapt their Web sites to new market demands and trends. Successful adaptation depends, among other things, on understanding users' needs. The Health on the Net Foundation (HON) has been conducting regular surveys of user-traffic since 1997. The fourth and latest in the series, conducted through the months of March and April, 1999, obtained 4,437 responses, compared to 1,863 responses obtained by the third survey in May and June, 1998. Thanks to these surveys, a broad user profile for Web-based medical and health-related information is emerging. The electronic questionnaire (in English, French, Italian and Portuguese) has remained largely the same since HON first posted it on its Web site in February-March, 1997. As in the past, results of the current survey are intended for use by all interested organisations. This article examines some major trends in user responses since then.

**Methods:**

The survey uses non-probabilistic sampling, and cannot ensure that participants are representative of the total medical and health information user community on the Web. Additional links to the HON survey posted by "friendly" Web sites (e.g. eBMJ and Intelihealth, USA, DoctorMiner (Brazil) and others helped boost participation. The questionnaire is designed for completion within three to four minutes. It starts with some basic user-related questions (including age, gender, location and years on the Internet). Next come a number of statements and a multiple choice of relevant answers, from "Strongly agree" to "Strongly disagree", for simple clicking with the mouse. HON added two new questions to the March-April, 1999 questionnaire: "What type of site do you first go to?", offering six multiple-choice options, and "What are your three most preferred Web sites?". The latter is the only open question in the survey. It requires users to provide their three "favourite" URLs. Respondents are also given an opportunity to leave spontaneous messages: in the last survey, 782 left a wide variety of remarks.

Results and Discussion:

The survey suggests an important growth in Internet use for medical and health-related information by users in Europe. Only 18% cannot find information in their primary language. The May-June, 1998 HON survey showed 71% of respondents were North American and only 18% European, while the March-April, 1999, survey showed 48% North American users and 28% Europeans. Further, most European users (79%) were medical professionals and male (67%), an important difference to North American users (49% medical professionals and 63% female).The survey suggests that, generally speaking, users are mature to middle-aged, relatively content with the variety of medical and health-related information on the Internet, increasingly multi-national in terms of country of origin, and increasingly concerned about quality.

